# Comparison of Viral Aerosol Shedding by Mild and Moderately Symptomatic Community‐Acquired and Nasally Inoculated Influenza A(H3) Infection

**DOI:** 10.1111/irv.70129

**Published:** 2025-06-13

**Authors:** Jianyu Lai, P. Jacob Bueno de Mesquita, Filbert Hong, Tianzhou Ma, Benjamin J. Cowling, Donald K. Milton

**Affiliations:** ^1^ Department of Global, Environmental, and Occupational Health University of Maryland School of Public Health College Park Maryland USA; ^2^ Department of Public Health Roger Williams University Bristol Rhode Island USA; ^3^ Department of Epidemiology and Biostatistics University of Maryland School of Public Health College Park Maryland USA; ^4^ World Health Organization Collaborating Center for Infectious Disease Epidemiology and Control, School of Public Health The University of Hong Kong Pok Fu Lam Hong Kong Special Administrative Region China

**Keywords:** aerosols, infection transmission, influenza, viral shedding

## Abstract

**Background:**

Nasally inoculated influenza cases reported milder symptoms and shed lower viral RNA load in exhaled breath aerosols (EBA) than people with classic influenza‐like illness in a previous study. Whether nasally inoculated influenza is representative of mild natural influenza infection is unknown. We extend previous analyses to include a broader range of community‐acquired cases.

**Methods:**

We previously studied (A) volunteers intranasally inoculated with a dose of 5.5 log_10_TCID_50_ of influenza A/Wisconsin/67/2005 (H3N2) and (B) cases with classic influenza‐like illness including fever recruited in 2013. We now add (C) cases from a 2017–2019 surveillance cohort of college dormitory residents and their contacts and (D) cases from a university health center in 2019. All cases had an influenza A(H3) infection. We collected 30‐min EBA samples using a Gesundheit‐II sampler.

**Results:**

Community‐acquired cases from the surveillance cohort (C) shed more EBA viral RNA and were more symptomatic than the inoculated cases (A) but shed less viral RNA than the symptom‐selected natural cases (B) from 2013, but not (D) from 2019. Despite similar symptoms to the 2013 selected cases (B), the 2019 community‐acquired cases (D) recruited post‐infection had lower fine aerosol viral RNA.

**Conclusions:**

Nasal inoculation of influenza virus did not reproduce EBA viral RNA shedding or symptoms observed in mild natural infection. Circulating strains of influenza A(H3) may differ year‐to‐year in the extent to which symptomatic cases shed virus into fine aerosols. New models, including possibly aerosol inoculation, are needed to study viral aerosol shedding from the human respiratory tract.

## 1 Introduction

Seasonal influenza virus infections impose a tremendous health burden [1, 2, 3, 4]. In the United States alone, the Centers for Disease Control and Prevention estimated that influenza‐related deaths ranged from 12,000 to 52,000 per season from 2010 to 2020, with between 140,000 and 710,000 hospitalizations per season [5]. Among the influenza strains, A(H3N2) viruses often result in more severe disease burdens in terms of morbidity and mortality compared to other strains, such as A(H1N1) and B viruses [2].

Existing research demonstrates the major role of aerosol inhalation in influenza transmission [6, 7]. One study estimated that approximately half of influenza transmissions in households occur via inhalation of aerosols [6]. Experimental influenza cases infected via intranasal inoculation of viruses typically shed lower levels of fine and coarse EBA than naturally‐infected symptomatic influenza cases [7, 8] and experience milder symptoms compared to those infected naturally [8, 9] or via aerosol inoculation [10]. Influenza A viral shedding quantity typically aligns with the progression of clinical symptoms over the course of the disease [8, 11].

Our previous comparison of viral shedding between natural and intranasally inoculated influenza cases primarily used naturally infected cases of acute respiratory illness, selected on the basis of having an objective fever or a positive rapid antigen test [8, 9]. This selection led to a lack of data on those with mild or asymptomatic influenza viral infection. Additionally, there has been limited comparative research on EBA viral shedding trajectories for both natural cases unselected for symptoms and experimental nasal inoculated cases. Despite observations that nasally inoculated cases shed less virus in EBA than naturally infected cases, there is a lack of overlap in symptom severity between the two groups, with the experimentally infected cases mostly presenting with mild illness or asymptomatically [8, 9].

This study sought to address these limitations by broadening the scope of prior analyses. We included unselected community‐acquired influenza cases from a surveillance cohort of college dormitory residents and their contacts. This group was defined as “unselected” due to being enrolled and followed up for cold or flu‐like symptoms regularly, with validated incentives for consistent responses [12]. We also included cases recruited post infections from a university health center, who generally presented with more symptoms. We compared these two groups with volunteers intranasally inoculated with influenza A/Wisconsin/67/2005(H3N2) as well as selected naturally infected influenza cases with cough and sore throat plus fever or a positive rapid antigen test recruited in 2013 that were reported previously [8]. Our aim was to examine the EBA viral shedding in an unselected sample of community‐acquired influenza cases and compare the EBA viral RNA loads and their trajectories between nasally inoculated cases and different community‐acquired influenza cases.

## 2 Methods

### 2.1 Data Sources and Sample Groups

This study used data obtained from three studies. The first two of these studies—Evaluating Modes of Influenza Transmission challenge study (EMIT‐1 experimental study) [13] and an observational study of selected acute respiratory illness (ARI) cases (EMIT‐1 campus study) [7]—have been previously reported [8]. The third study followed uninfected volunteers over an academic year for their ARI occurrence, known as the Prometheus study [12].

The EMIT‐1 experimental study was conducted in compliance with UK regulatory and ethical (IRB) requirements (under auspices of the UK Health Research Authority [HRA] National Research Ethics Service [NRES] Committee London‐City & East; reference number 12/LO/1277) and registered with ClinicalTrials.gov (number NCT01710111). The EMIT‐1 campus study and the Prometheus study were approved by the University of Maryland Institutional Review Board. Participants from all three sources have signed informed consent.

#### 2.1.1 EMIT‐1 Experimental Study

This study, initiated in 2013 to investigate questions related to influenza transmission [13], involved volunteers divided into two groups: “Donors” and “Recipients.” The Donors were intranasally inoculated with a dose of 5.5 log_10_TCID_50_ influenza A/Wisconsin/67/2005 and subsequently confirmed to have an influenza A infection (H3N2). After inoculation, the Donors were paired with uninfected healthy Recipients in a common room to encourage interaction [13]. For the purposes of this report, we exclusively included the Donors, representing experimental cases, and have designated them as Group A.

#### 2.1.2 EMIT‐1 Campus Study

The EMIT‐1 campus study was conducted at the University of Maryland, College Park (UMD) [7, 8]. Volunteers presenting symptoms of acute respiratory infections were recruited from December 2012 to March 2013. Those who eventually provided breath samples were within 3 days of symptom onset and had a positive QuickVue Influenza A + B test (Quidel, San Diego, CA) or were still febrile with an oral temperature of greater than 37.8°C plus coughing or a sore throat [7, 8]. In the context of our current study, we incorporated those with a positive nasopharyngeal swab for influenza A/H3N2 as confirmed by reverse transcription polymerase chain reaction (RT‐PCR). This group, representing the severe spectrum of symptoms, was classified as Group B.

#### 2.1.3 Prometheus Study

The Prometheus study was also conducted at the UMD community [12]. From 2017 to 2019, students and staff from UMD were actively recruited and monitored for the occurrence of acute respiratory infections during each academic year. Volunteers were instructed to report to the study team immediately upon developing cold or flu‐like symptoms. The identified volunteers and their close contacts were then invited to a research clinic to provide samples for further examination. For the 2018–2019 flu season, additional volunteers were recruited from the university health center, typically presenting with more severe symptoms than those recruited from the surveillance cohort. For this study, we included individuals with confirmed influenza A via mid‐turbinate or nasal and/or throat swabs, as identified by a TaqMan Array Card (Thermo Fisher, Waltham, MA, USA). Participants from the community, generally presenting with very mild symptoms, were assigned to Group C, while those recruited from the university health center were classified as Group D.

### 2.2 Sample Collection

For all these studies, volunteers with confirmed viral infections were invited to provide 30‐min EBA samples using a Gesundheit‐II sampler [14]. They were allowed to breathe normally and cough spontaneously during the collection. After collection, EBA samples were categorized by size into two fractions: fine aerosols (diameter ≤ 5 μm) and coarse aerosols (> 5 μm). Viral RNA was extracted from both fine and coarse aerosol samples and quantified using real‐time RT‐PCR.

### 2.3 Statistical Analyses

In our statistical analysis, we included only EBA samples on which day their nasal and/or throat swabs were positive.

Descriptive analysis was carried out for the four comparison groups (A, B, C, and D). We compared the four groups and the three natural infection groups using the Kruskal–Wallis test for the continuous variables and Fisher's exact test for the categorical variables. We also did pair‐wise comparison among the three natural infection groups, using T‐test for the continuous variables and Fisher's exact test or chi‐square test for the categorical variables.

To visualize change over time, we plotted the EBA viral RNA loads as well as symptom scores by study day for the four groups separately. The study day was defined as days post symptom onset for Groups B, C, and D and days post inoculation for Group A, as most of the experimental cases were asymptomatic or not sampled on the days after symptom onset. We then identified the incubation period (*I*) for Group A by comparing the peak of symptom scores in Group A and the other three groups.

Linear mixed effect models with censored responses (R package “lmec,” version 1.0 [15]) were used to calculate the geometric means (GM) of the EBA viral RNA load for the four comparison groups. These models accounted for the censored outcome variable (i.e., viral RNA load below limit of detection) as well as nested random effects of individuals and samples within the same individuals. We also used the lmec model to estimate the group effect on EBA viral shedding (Model I) and the shedding over time (Model II), controlling for potential confounders and effect modifiers. For Model I, age, sex, and study day (defined as day post symptom onset for Group B, C, and D, and day post inoculation minus *I* for Group A) were included into the models as potential confounders. Then the product terms of each of these three variables and the group variable were included for further interaction assessment. The best models were selected based on Akaike information criterion (AIC). For Model II, we forced the interaction terms of group and study day into the model along with age and sex and then further selected the final model based on AIC.

We conducted all analyses in R version 4.2.3 (R Foundation for Statistical Computing, Vienna, Austria) and RStudio.

## 3 Results

### 3.1 Characteristics of the Study Population

**FIGURE 1:**
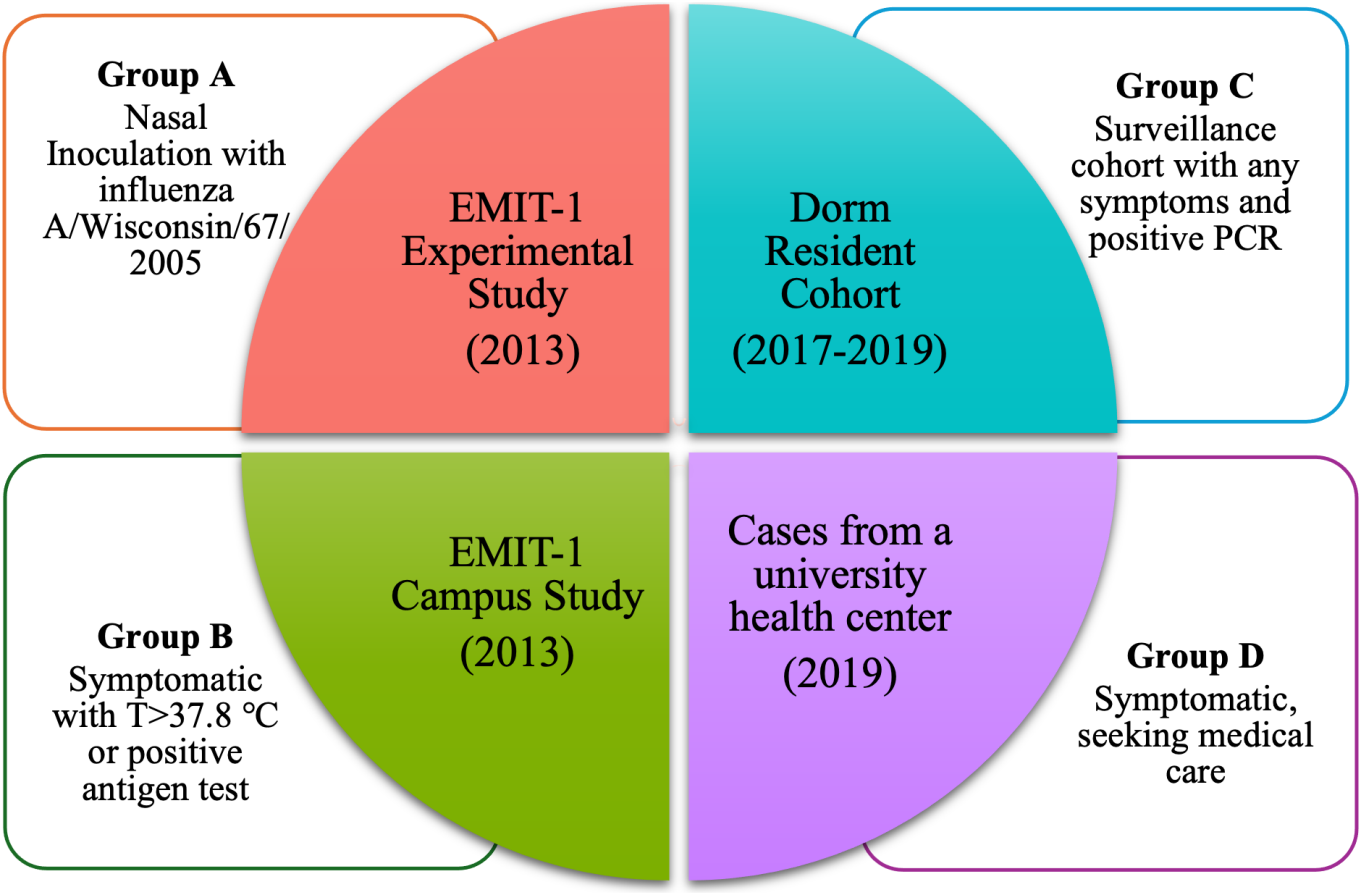
Study population.

This study included a total of 143 participants infected with influenza A(H3), with 36 nasally inoculated with influenza A/Wisconsin/67/2005(H3N2) (Group A), 83 selected symptomatic cases with fever or a positive antigen test recruited in 2013 (Group B), 17 unselected cases recruited from the surveillance dorm resident cohort (Group C), and 7 symptomatic cases recruited from a university health center (UHC) in 2019 (Group D) (Figure 1). Group A had the most participants over 25 years of age (mean age: 30.1 years), while the other three groups predominantly consisted of younger adults, aged 18–25 years. A higher proportion of females was found in Groups B (57%) and C (65%) compared to Groups A (31%) and D (29%) (Tables 1 and S1).

**TABLE 1:** Characteristics of the study population.

	A: Nasal inoculation with influenza A/Wisconsin/67/2005	B: Symptomatic with T > 37.8°C or positive antigen test	C: Surveillance cohort with any symptoms and positive PCR	D: Symptomatic, seeking medical care	All participants
Number of participants	36	83	17	7	143
Influenza Season, *N* (%)					
2012–2013	36 (100)	83 (100)	0 (0)	0 (0)	119 (83)
2016–2017	0 (0)	0 (0)	3 (18)	0 (0)	3 (2)
2017–2018	0 (0)	0 (0)	4 (24)	0 (0)	4 (3)
2018–2019	0 (0)	0 (0)	10 (59)	7 (100)	17 (12)
Female, *N* (%)	11 (31)	47 (57)	11 (65)	2 (29)	71 (50)
Age, mean ± SD	30.1 ± 7.2	22.3 ± 7.6	19.3 ± 0.9	20 ± 1.4	23.8 ± 7.8
Age group, *N* (%)					
< 18	0 (0)	2 (2)	0 (0)	0 (0)	2 (1)
18–25	11 (31)	73 (88)	17 (100)	7 (100)	108 (76)
> 25	25 (69)	8 (10)	0 (0)	0 (0)	33 (23)
With fever > 37.9°C, *N* (%)^a^	1 (3)	17 (20)	2 (12)	1 (14)	21 (15)
Coughs per 30 min, mean ± SD (range)^b^	2 ± 6 (0–35)	27 ± 33 (0–265)	13 ± 18 (0–66)	15 ± 21 (0–60)	19 ± 29 (0–265)
Temperature (C), mean ± SD^c^	36.6 ± 0.6	37.3 ± 0.6	37.3 ± 0.8	37.6 ± 0.7	37.2 ± 0.7
Median upper respiratory symptoms (IQR)^d^	1 (0–3)	7 (5–9)	6 (4–7)	5 (3.5–6.5)	5 (3–8)
Median lower respiratory symptoms (IQR)	0 (0–0)	3 (2–4)	3 (2–3)	2 (2–4.5)	2 (1–4)
Median systemic symptoms (IQR)	0 (0–1)	5 (4–7)	3 (1–6)	6 (4–7.5)	4 (1–6.5)

^a^ Fever at the time of breath sample collection. Tympanic temperature was measured for Group A, and oral temperature was measured for Groups B, C, and D. One subject in Group C did not have body temperature measured.

^b^ Cough counts were missing for four observations in Group A, three observations in Group B, and three observations in Group C.

^c^ Temperature was missing for one observation in each of Groups A, B, and C.

^d^ Upper respiratory symptoms (range 0–15): runny nose, stuffy nose, sneezing, sore throat, and earache symptom scores; lower respiratory symptoms (range 0–6): shortness of breath and cough; systemic symptoms (range 0–9): malaise, headache, and muscle/join ache.

As previously reported [8], Group A had very mild or non‐existent symptoms. This group had the lowest median score for all three symptoms—upper respiratory symptoms, lower respiratory symptoms, and systemic symptoms. They also had the lowest incidence of febrile cases (3%) and a reduced frequency of coughs at the time of sampling. Group C, the unselected community‐acquired cases from the surveillance cohort, demonstrated a medium level of symptom severity across the groups. Specifically, we found a significant difference among the three community‐acquired infection groups in terms of their systemic symptoms and a borderline significant difference for upper respiratory symptoms (Table S1). Group C had significantly lower upper respiratory and systemic symptom scores than Group B. For Group B and D, the symptom distribution, as well as other characteristics reported, did not demonstrate substantial differences (Tables 1 and S1 and Figure S1).

### 3.2 Positive Detection Rates and Geometric Means of EBA Viral Shedding

Group A demonstrated a significantly lower rates of positive detection for both coarse and fine EBA, in terms of the number of positive samples and positive cases, than Group B and D. Group C had a slightly higher proportion of positive cases and samples than Group A, although the differences were not significant.

There was no significant difference between Groups B and D in the proportions of positive cases and positive EBA samples. When comparing to Group C, we only found a significant difference in the rates of positive cases and samples for fine EBA between Groups B and C (Table 2).

**TABLE 2:** Viral shedding into exhaled breath aerosols.

	A: Nasal inoculation with influenza A/Wisconsin/67/2005		B: Symptomatic with T > 37.8°C or positive antigen test		C: Surveillance cohort with any symptoms and positive PCR		D: Symptomatic, seeking medical care	
Year	2013		2013		2017–2019		2019	
EBA	Coarse	Fine	Coarse	Fine	Coarse	Fine	Coarse	Fine
Cases	36	36	83	83	17	17	7	7
Samples	64	64	143	143	21	21	7	7
No. of positive cases (%)	6 (17)	11 (31)	43 (52)	71 (86)	5 (29)	8 (47)	4 (57)	6 (86)
No. of positive samples (%)	6 (9)	14 (22)	64 (45)	110 (77)	5 (24)	8 (38)	4 (57)	6 (86)

**FIGURE 2:**
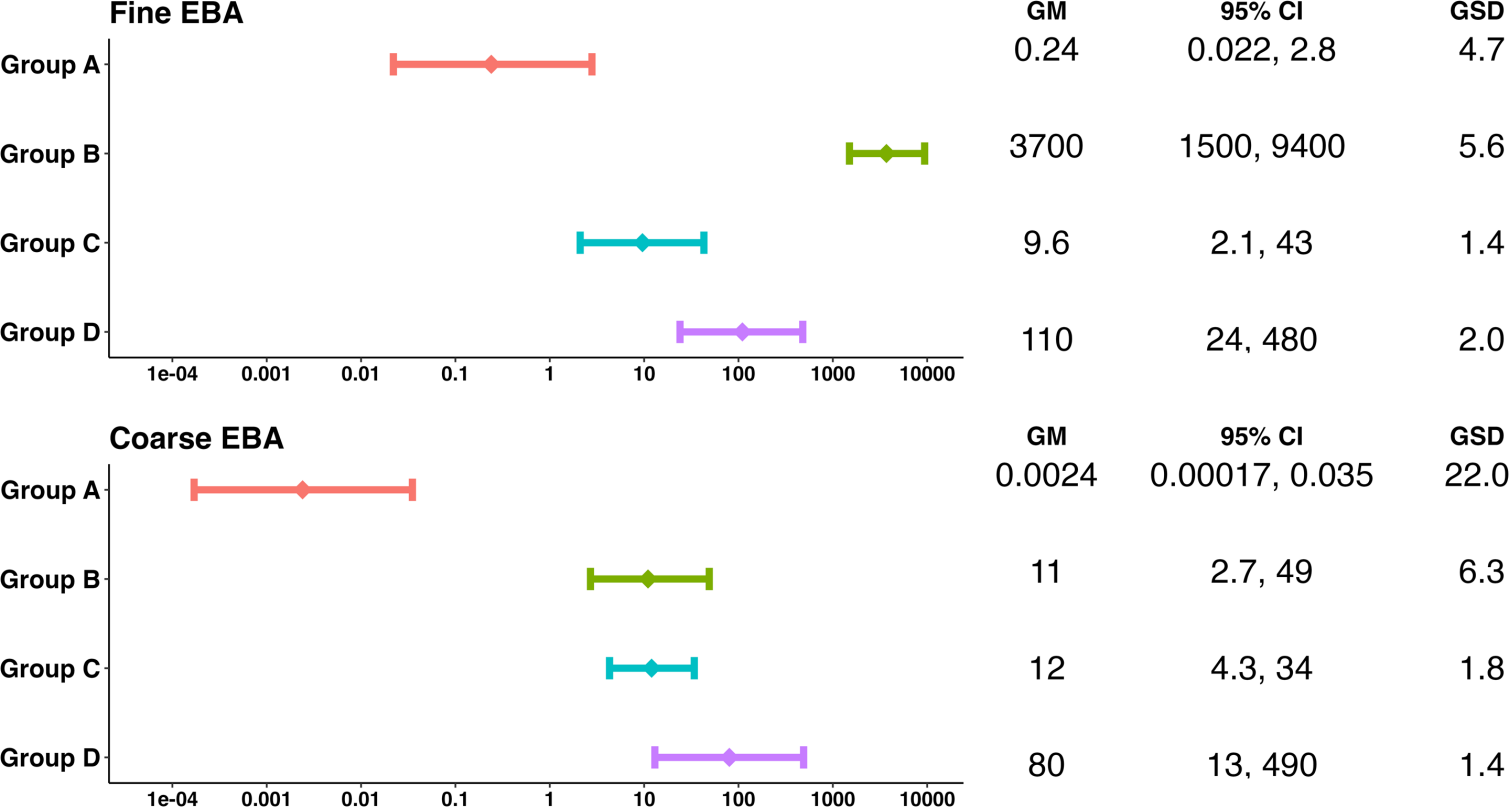
Geometric means of EBA viral shedding for the four groups. GM, geometric means; GSD, geometric standard deviation. Group A: nasal inoculation with influenza A/Wisconsin/67/2005; Group B: symptomatic with T > 37.8°C or positive antigen test; Group C: surveillance cohort with any symptoms and positive PCR; Group D: symptomatic, seeking medical care. The GM and GSD were computed for all the samples using linear mixed‐effects models for censored responses (R Project package “lmec”). These models accounted for the censored outcome variable as well as nested random effects of individuals and samples within the same individuals.

Figure 2 shows geometric mean viral RNA copy numbers measured in aerosols collected per 30‐mimute sampling period. In this analysis without controlling for confounders, Group A had a significantly lower geometric mean viral RNA copy numbers in both fine and coarse EBA, relative to the other three groups (Figure 2). Group B presented a significantly higher geometric mean viral RNA copy numbers in fine, but not in coarse EBA, compared to Groups C and D. Both Groups C and D displayed intermediate levels of fine EBA viral RNA load, although D had a higher value than C.

### 3.3 Temporal Patterns of Symptoms and EBA Viral RNA Shedding

**FIGURE 3:**
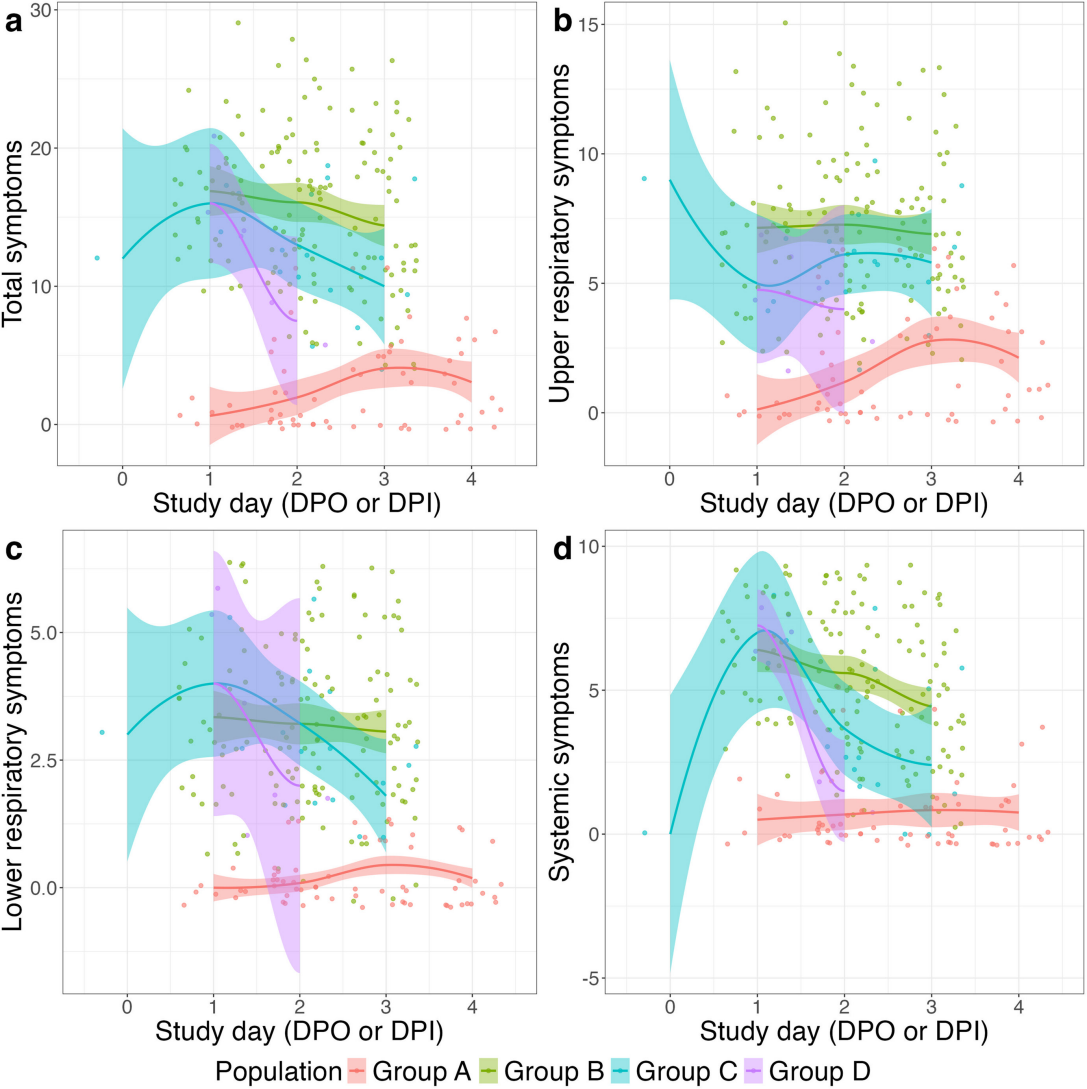
Trajectories of symptom scores over study days. Group A: nasal inoculation with influenza A/Wisconsin/67/2005; Group B: symptomatic with T > 37.8°C or positive antigen test; Group C: surveillance cohort with any symptoms and positive PCR; Group D: symptomatic, seeking medical care. Study day was defined as day post symptom onset for Groups B, C, and D and day post inoculation for Group A.

For Groups B, C, and D, symptom scores peaked on the first day following symptom onset, with the exception of upper respiratory symptom scores in Group C, which fluctuated post‐onset. For Group A, all symptom scores reached their peak on the third day post‐inoculation. Based on the observed trajectories, we estimate an incubation period of 2 days for Group A (Figure 3).

**FIGURE 4:**
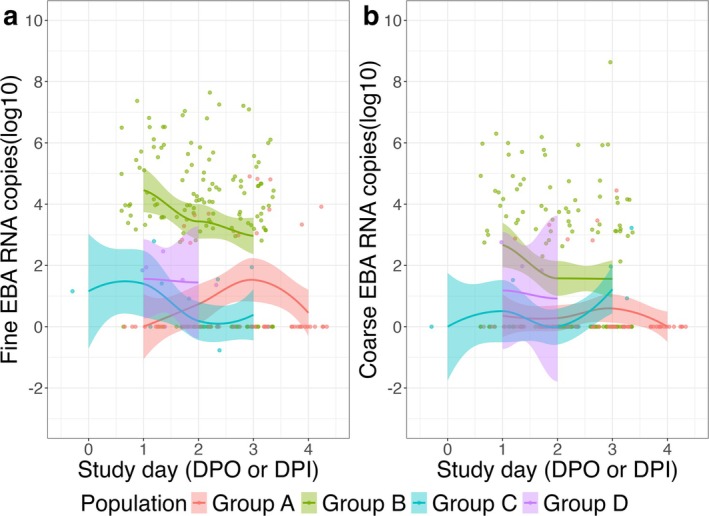
Trajectories of EBA viral shedding over study days. Group A: nasal inoculation with influenza A/Wisconsin/67/2005; Group B: symptomatic with T > 37.8°C or positive antigen test; Group C: surveillance cohort with any symptoms and positive PCR; Group D: symptomatic, seeking medical care. Study day was defined as day post symptom onset for Groups B, C, and D and day post inoculation for Group A.

The trajectories of EBA viral RNA shedding mirrored those of the symptom scores, with the exception of Group C. For this group, the EBA shedding trajectory followed the symptom trajectory for the initial 2 days but then increased on the third day post‐symptom onset (Figure 4).

### 3.4 Modeling Group Effects on EBA Viral RNA Shedding

**FIGURE 5:**
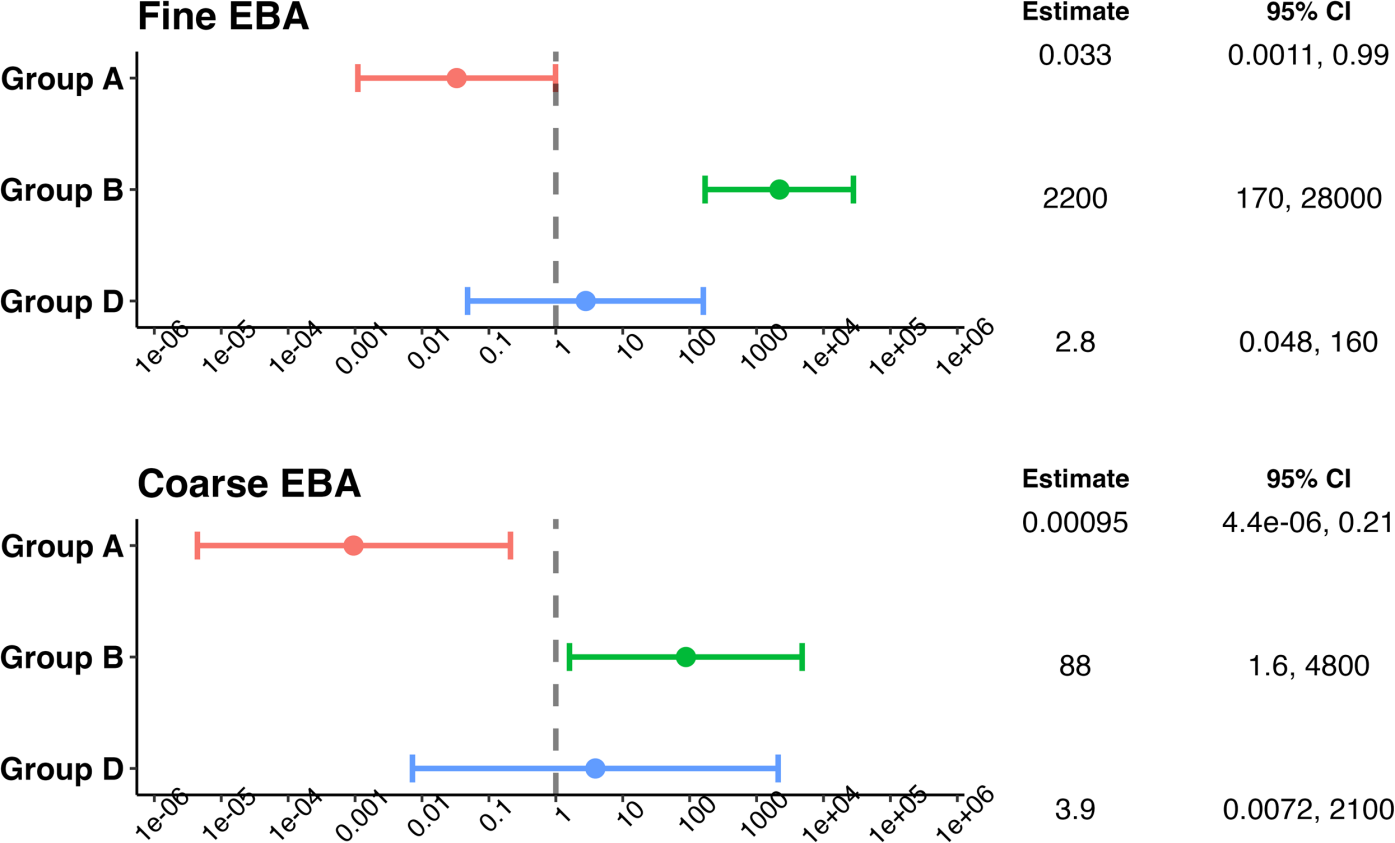
The ratio of viral shedding in EBA of selected and inoculated cases to shedding by unselected cases (Group C), controlling for age, sex, and study days. Group A: nasal inoculation with influenza A/Wisconsin/67/2005; Group B: symptomatic with T > 37.8°C or positive antigen test; Group C: surveillance cohort with any symptoms and positive PCR; Group D: symptomatic, seeking medical care. We included only those samples whose study day was not missing (three C and one D cases were excluded due to not having symptom onset date on file or having no symptoms over the course of the follow‐up period). We used linear mixed‐effects models for censored responses (R Project package “lmec”) to estimate the ratio of viral shedding in EBA of Group A/B/D to Group C. These models accounted for the censored outcome variable as well as nested random effects of individuals and samples within the same individuals. The best models were selected based on Akaike information criterion (AIC).

In models controlling for potential confounding variables and effect modifiers (age, sex, and study days), Group A continued to present a significantly lower viral RNA load in both aerosol fractions. The ratio of viral RNA shedding of Group A to that of Group C was the lowest, with a value of 3.3 × 10^−2^ (95% CI: 1.1 × 10^−3^, 0.99) for fine aerosol and 9.5 × 10^−4^ (95% CI: 4.4 × 10^−6^, 0.21) for coarse aerosol. Group B, on the other hand, had a significantly higher viral RNA load in both aerosols compared to Group C, with the ratio as 2.2 × 10^3^ (95% CI: 1.7 × 10^2^, 2.8 × 10^4^) for fine and 88 (95% CI: 1.6, 4.8 × 10^3^) for coarse aerosols. There was no significant difference between Groups C and D in terms of viral RNA load in both EBA size fractions (Figure 5 and Table S2). Contrast analysis revealed that Group B had a significantly higher viral load in fine aerosols than Group D, although no significant difference was found for coarse aerosols (Table S3).

Regarding shedding trajectories over time, no significant difference was observed between Group C and the rest of the groups concerning the dynamics of EBA viral RNA shedding by study days (Table S4).

## 4 Discussion

This study provides a unique comparison of EBA viral shedding patterns among influenza A/H3 infected individuals in distinct groups: nasally inoculated cases, unselected community‐acquired cases from a surveillance cohort, and selected natural cases from different influenza seasons. We found previously that selected natural cases shed higher viral loads in EBAs and were more symptomatic than experimental cases [8]. This study further adds that unselected community‐acquired cases, representing a wider spectrum of symptom severity, shed more viral RNA in their exhaled breath aerosols and were more symptomatic than the experimental nasally inoculated cases as well, but, on average, shed less viral RNA than the selected natural cases that were more symptomatic and from different influenza seasons.

Those inoculated nasally with a high dose of influenza virus A/Wisconsin/67/2005 strain had a high percentage of people who did not have detectable levels of viral RNA load in their exhaled breath aerosols or have any symptoms, despite shedding substantial virus into nasopharyngeal swabs. This indicates that nasal inoculation with this strain may not provide a robust model for understanding EBA viral shedding patterns or the range of symptom severity typical in natural influenza virus infections. Our findings are consistent with prior research indicating that intranasal inoculation with influenza often results in milder symptoms compared to both natural infection and inhalation of infectious particles [10]. Little et al. [9] compared experimental influenza cases to those with naturally acquired influenza and found that the former group presented with milder illness and shorter cough duration. Henle et al. [16] demonstrate that inhalation of various strains of influenza viruses led to febrile in human subjects, a response rarely seen with intranasal instillation. Given the major role of aerosol inhalation in influenza transmission [6, 7], inoculation via aerosol inhalation might offer a more representative model for mimicking natural influenza infections [17]. In addition, animal studies have demonstrated that aerosol inhalation of recombinant influenza viruses results in a more efficient infection of the lower respiratory tract and faster viral replication compared to intranasal inoculation [18]. This might explain the lower viral load found in the exhaled breath aerosols of individuals inoculated intranasally, as opposed to those infected naturally.

Of note, despite sharing similar characteristics and symptomatic profiles, the two groups of medically attended cases from different years (Groups B and D) exhaled statistically different fine aerosol viral RNA loads. The discrepancy in the relationship between viral shedding and symptoms could be related to year‐to‐year differences in circulating strains of influenza A(H3). The elevated viral load in fine aerosols observed in participants from the 2012–2013 influenza season might, in part, explain the heavier influenza burden during that season compared to the 2018–2019 influenza season in the United States [5]. While our sample size and study design limit definitive conclusions, these differences raise important questions about how seasonal variability may influence transmission dynamics. Future research is needed to confirm these findings and evaluate their potential to inform infection control strategies.

This study had several strengths. We did a unique comparison of EBA viral shedding pattern among influenza A(H3) infected individuals from three sources, allowing us to compare the viral shedding between experimental infections and the full range of ambulatory community‐acquired influenza virus infections. Including participants from multiple years also allowed us to begin to explore year‐to‐year variation in shedding likely driven by a combination of virus strains, immunity, and vaccine effectiveness.

Our research was subject to some limitations. Our study volunteers were all ambulatory; hence, the results of this study may not be generalizable to those who are critically ill. We did not have culture results for all the EBA samples; hence, we could not directly compare infectious viral load and potentially the risk of inhalation transmission among the groups. Despite this, previous work reported a correlation between viral RNA and quantitative culture [7]. Another limitation of this study is the inherent difference between naturally acquired and experimentally induced influenza infections. While we attempted to align the timing of symptom onset across groups to account for the unknown incubation period for the community‐acquired cases, the exact timing, dose, and mode of exposure in community‐acquired infections remain unknown. These factors may also contribute to differences in viral shedding patterns.

This study found that participants experimentally infected via nasal inoculation presented with milder symptoms and lower levels of exhaled breath aerosol viral shedding compared to those with a wider range of naturally acquired influenza. These findings, along with previous work showing that intranasal inoculation often fails to produce the full spectrum of influenza‐like illness, highlight the limitation of current challenge models. They also suggest the need for alternative approaches to study viral aerosol shedding from the human respiratory tract. In particular, aerosol inoculation may represent a promising avenue for future research, as it could better mimic the natural infection process and offer insights into aerosol transmission dynamics. Overall, this study contributes to the broader understanding of viral shedding patterns in EBA and underscores the importance of developing robust models to further our understanding of influenza transmission.

## Author Contributions

**Jianyu Lai:** conceptualization, methodology, data curation, writing – original draft, writing – review and editing, investigation, formal analysis, visualization. **P. Jacob Bueno de Mesquita:** conceptualization, methodology, data curation, writing – review and editing, investigation. **Filbert Hong:** data curation, writing – review and editing, investigation, project administration. **Tianzhou Ma:** methodology, writing – review and editing. **Benjamin J. Cowling:** methodology, writing – review and editing, investigation. **Donald K. Milton:** conceptualization, methodology, writing – review and editing, investigation, funding acquisition, project administration, resources, supervision.

## Ethics Statement

The EMIT‐1 experimental study was conducted in compliance with UK regulatory and ethical (IRB) requirements (under auspices of the UK Health Research Authority [HRA] National Research Ethics Service [NRES] Committee London‐City & East; reference number 12/LO/1277) and registered with ClinicalTrials.gov (number NCT01710111). The EMIT‐1 campus study and the Prometheus study were approved by the University of Maryland Institutional Review Board.

## Consent

Participants from all three sources have signed informed consent.

## Conflicts of Interest

B.J.C. consults for AstraZeneca, Fosun Pharma, GlaxoSmithKline, Haleon, Moderna, Novavax, Pfizer, Roche, and Sanofi Pasteur. D.K.M. consults for A.I.R LLC and holds stock options for Lumen Bioscience Inc. The other authors declare no conflicts of interest.

## Peer Review

The peer review history for this article is available at https://www.webofscience.com/api/gateway/wos/peer‐review/10.1111/irv.70129.

## Supporting information


**Table S1** Group comparisons for the characteristics of the study population. ^†^Four‐group and three‐group comparison: Kruskal–Wallis test for the continuous variables and Fisher’s exact test for the categorical variables. ^‡^Two‐group comparison: T‐test for the continuous variables and Fisher’s exact test or chi‐square test for the categorical variables.
**Table S2.** The effect of group (ref = Group C) on EBA viral RNA shedding.^†,‡ †^Group A: nasal inoculation with influenza A/Wisconsin/67/2005; Group B: symptomatic with T > 37.8°C or positive antigen test; Group C: surveillance cohort with any symptoms and positive PCR; Group D: symptomatic, seeking medical care. ^‡^We used linear mixed‐effects models for censored responses (R Project package “lmec”) to estimate the ratio of viral shedding in EBA of Group A/B/D to Group C. These models accounted for the censored outcome variable as well as nested random effects of individuals and samples within the same individuals. The best models were selected based on Akaike information criterion (AIC). ^§^Study day was defined as day post symptom onset for Groups B, C, and D and day post inoculation minus 2 days for Group A. We included only those samples whose study day was not missing (three C and one D cases were excluded due to not having symptom onset date on file or having no symptoms over the course of the follow‐up period).
**Table S3.** Contrast analysis on the relative effect of selected and inoculated cases.^† †^Group A: nasal inoculation with influenza A/Wisconsin/67/2005; Group B: symptomatic with T > 37.8°C or positive antigen test; Group D: symptomatic, seeking medical care.
**Table S4.** The effect of group (ref = Group C) on EBA viral RNA shedding over study day.^†,‡,§ †^Group A: nasal inoculation with influenza A/Wisconsin/67/2005; Group B: symptomatic with T > 37.8°C or positive antigen test; Group C: surveillance cohort with any symptoms and positive PCR; Group D: symptomatic, seeking medical care. ^‡^We used linear mixed‐effects models for censored responses (R Project package “lmec”) to model the effect of groups on viral shedding over time. These models accounted for the censored outcome variable as well as nested random effects of individuals and samples within the same individuals. The best models were selected based on Akaike information criterion (AIC). None of the interaction terms demonstrated a significant effect. ^§^Study day was defined as day post symptom onset for Groups B, C, and D and day post inoculation minus 2 days for Group A. We included only those samples whose study day was not missing (three C and one D cases were excluded due to not having symptom onset date on file or having no symptoms over the course of the follow‐up period).
**Figure S1.** Mean symptom scores, body temperatures, and cough counts across groups. Group A: nasal inoculation with influenza A/Wisconsin/67/2005; Group B: symptomatic with T > 37.8°C or positive antigen test; Group C: surveillance cohort with any symptoms and positive PCR; Group D: symptomatic, seeking medical care.

## Data Availability

Deidentified data and custom code used to analyze the data can be accessed on the Open Science Framework repository: https://osf.io/3z5ny/?view_only=6a5de070c88e452990a886a67cb8544f.
