# Supplementary material for: Comparison of Viral Aerosol Shedding by Mild and Moderately Symptomatic Community‐Acquired and Nasally Inoculated Influenza A(H3) Infection
Source: Influenza Other Respir Viruses. 2025 Jun 13;19(6):e70129. doi: 10.1111/irv.70129 (PMC12163343; doi:10.1111/irv.70129)
Supplement: Supplementary file 1 — Table S1 Group comparisons for the characteristics of the study population. †Four‐group and three‐group comparison: Kruskal–Wallis test for the continuous variables and Fisher’s exact test for the categorical variables. ‡Two‐group comparison: T‐test for the continuous variables and Fisher’s exact test or chi‐square test for the categorical variables. Table S2. The effect of group (ref = Group C) on EBA viral RNA shedding.†,‡ †Group A: nasal inoculation with influenza A/Wisconsin/67/2005; Group B: symptomatic with T > 37.8°C or positive antigen test; Group C: surveillance cohort with any symptoms and positive PCR; Group D: symptomatic, seeking medical care. ‡We used linear mixed‐effects models for censored responses (R Project package “lmec”) to estimate the ratio of viral shedding in EBA of Group A/B/D to Group C. These models accounted for the censored outcome variable as well as nested random effects of individuals and samples within the same individuals. The best models were selected based on Akaike information criterion (AIC). §Study day was defined as day post symptom onset for Groups B, C, and D and day post inoculation minus 2 days for Group A. We included only those samples whose study day was not missing (three C and one D cases were excluded due to not having symptom onset date on file or having no symptoms over the course of the follow‐up period). Table S3. Contrast analysis on the relative effect of selected and inoculated cases.† †Group A: nasal inoculation with influenza A/Wisconsin/67/2005; Group B: symptomatic with T > 37.8°C or positive antigen test; Group D: symptomatic, seeking medical care. Table S4. The effect of group (ref = Group C) on EBA viral RNA shedding over study day.†,‡,§ †Group A: nasal inoculation with influenza A/Wisconsin/67/2005; Group B: symptomatic with T > 37.8°C or positive antigen test; Group C: surveillance cohort with any symptoms and positive PCR; Group D: symptomatic, seeking medical care. ‡We used linear mixed‐effects mod [file IRV-19-e70129-s001.docx]

**Supplementary materials**

**Table of Content**

[EMIT Consortium Team Members 2](#_Toc177949427)

[Prometheus-UMD investigators 3](#_Toc177949428)

[Supplementary Tables 5](#_Toc177949429)

[Supplementary Table 1. Group comparisons for the Characteristics of the study population 5](#_Toc177949430)

[Supplementary Table 2. The effect of group (ref = Group C) on EBA viral RNA shedding 7](#_Toc177949431)

[Supplementary Table 3. Contrast analysis on the relative effect of selected and inoculated cases 8](#_Toc177949432)

[Supplementary Table 4. The effect of group (ref = Group C) on EBA viral RNA shedding over study day 9](#_Toc177949433)

[Supplementary Figure 11](#_Toc177949434)

[Supplementary Figure 1. Mean symptom scores, body temperatures, and cough counts across groups. 11](#_Toc177949435)

## EMIT Consortium Team Members

EMIT team members were: Walt Adamson, Blanca Beato-Arribas, Werner Bischoff, William Booth, Simon Cauchemez, Sheryl Ehrman, Joanne Enstone, Neil Ferguson, John Forni, Anthony Gilbert, Michael Grantham, Lisa Grohskopf, Andrew Hayward, Michael Hewitt, Ashley Kang, Ben Killingley, Robert Lambkin-Williams, Alex Mann, Donald Milton, Jonathan Nguyen-Van-Tam, Catherine Noakes, John Oxford, Massimo Palmarini, Jovan Pantelic, and Jennifer Wang. The Scientific Advisory Board members were: Allan Bennett, Ben Cowling, Arnold Monto, and Raymond Tellier.

## Prometheus-UMD investigators

Addo, Kofi

Adenaiye, Oluwasanmi Oladapo

Agrawala, Agrawala

Aiello, Allison

Albert, Barbara

Arria, Amelia

Bueno de Mesquita, P. Jacob

Cai, Mara

Chen, Shuo

Chen, Wilbur

Corrada Bravo, Hector

Elworth, Leo

Felgner, Philip

Frieman, Matthew

German, Jennifer

Heidarinejad, Mohammad

Hong, Filbert

Jiang, Chengsheng

Khan, Saahir

Lai, Jianyu

Liu, Hongjie

Ma, Tianzhou

Maljkovic Berry, Irina

Martinello, Richard

Mattise, Nick

Memon, Atif

Milton, Donald

Mongodin, Emmanuel

Nasko, Dan

Pop, Mihai

Porter, Adam

Romo, Sebastian

Srebric, Jelena

Tai, Sheldon

Treangen, Todd

Wajid, Faizan

Washington-Lewis, Rhonda

Wu, Qiong

Xing, Yishi

Youssefi, Somayeh

Zhu, Shengwei

## Supplementary Tables

### **Supplementary Table 1. Group comparisons for the Characteristics of the study population**

|  | 4-group comparison^†^ (A, B, C, D),  *p* | 3-group comparison (B, C, D),  *p* | C vs. D^‡^,  *p* | B vs. C,  *p* | B vs. D,  *p* |
| --- | --- | --- | --- | --- | --- |
| Flu season | <0.001 | <0.001 | 0.222 | <0.001 | <0.001 |
| Female | 0.02 | 0.293 | 0.182 | 0.73 | 0.239 |
| Age | <0.001 | 0.008 | 0.159 | <0.001 | 0.433 |
| Age group | <0.001 | 0.695 | 1 | 0.549 | 1 |
| With fever > 37.9°C | 0.056 | 0.896 | 1 | 0.73 | 1 |
| Coughs per 30 min | <0.001 | 0.042 | 0.826 | 0.08 | 0.338 |
| Temperature (C) | <0.001 | 0.14 | 0.32 | 0.711 | 0.256 |
| Upper respiratory symptoms | <0.001 | 0.061 | 0.87 | 0.028 | 0.139 |
| Lower respiratory symptoms | <0.001 | 0.403 | 0.498 | 0.146 | 0.939 |
| Systemic symptoms | <0.001 | 0.004 | 0.08 | <0.001 | 0.957 |

†. Four-group and three-group comparison: Kruskal-Wallis test for the continuous variables and Fisher’s exact test for the categorical variables

‡. Two-group comparison: T-test for the continuous variables and Fisher’s exact test or Chi-square test for the categorical variables.

**Supplementary Table 2. The effect of group (ref = Group C) on EBA viral RNA shedding**^†,^ ^‡^

| Variables | | Fine EBA | Coarse EBA |
| --- | --- | --- | --- |
| Group^‡^(Ref=C) | A | 0.033 (0.0011, 0.99) | 0.00095 (4.4e-06, 0.21) |
|  | B | 2200 (170, 28000) | 88 (1.6, 4800) |
|  | D | 2.8 (0.048, 160) | 3.9 (0.0072, 2100) |
| Study Day^§^  (DPS/DPI-2) | | 0.27 (0.14, 0.52) | 0.19 (0.079, 0.46) |
| Age | | 1.1 (0.95, 1.2) | 1.1 (0.94, 1.3) |
| Sex | | 8.2 (1.8, 36) | 6.4 (0.65, 62) |

†. Group A: Nasal Inoculation with influenza A/Wisconsin/67/2005; Group B: Symptomatic with T>37.8˚C or positive antigen test; Group C: Surveillance cohort with any symptoms and positive PCR; Group D: Symptomatic, seeking medical care.

‡. We used linear mixed-effects models for censored responses (R Project package “lmec”) to estimate the ratio of viral shedding in EBA of Group A/B/D to Group C. These models accounted for the censored outcome variable as well as nested random effects of individuals and samples within the same individuals. The best models were selected based on Akaike Information Criterion (AIC).

§. Study Day was defined as day post symptom onset for Group B, C, and D, and day post inoculation minus two days for Group A. We included only those samples whose study day was not missing (three C and one D cases were excluded due to not having symptom onset date on file or having no symptoms over the course of the follow-up period).

**Supplementary Table 3. Contrast analysis on the relative effect of selected and inoculated cases**^†^

|  | Fine EBA | | | Coarse EBA | | |
| --- | --- | --- | --- | --- | --- | --- |
|  | Contrast coefficient | Standard error | p-value | Contrast coefficient | Standard error | p-value |
| Group A vs. Group B | -11 | 1.2 | <0.001 | -11 | 1.9 | 4.3 x 10^-9^ |
| Group A vs. Group D | -4.4 | 2 | 0.025 | -8.3 | 3.1 | 0.007 |
| Group D vs. Group B | -6.7 | 1.7 | 0.00012 | -3.1 | 2.7 | 0.24 |

†. Group A: Nasal Inoculation with influenza A/Wisconsin/67/2005; Group B: Symptomatic with T>37.8˚C or positive antigen test; Group D: Symptomatic, seeking medical care.

**Supplementary Table 4. The effect of group (ref = Group C) on EBA viral RNA shedding over study day^†,‡,§^**

| Variables | | Fine EBA | Coarse EBA |
| --- | --- | --- | --- |
| Group (Ref=C) | A | 0.0052 (1.7e-05, 1.6) | 3.7 (4.6e-05, 310000) |
|  | B | 1200 (4.9, 280000) | 720000 (13, 4e+10) |
|  | D | 0.25 (4.5e-06, 14000) | 11000 (0.00017, 6.5e+11) |
| Study Day (DPS/DPI-2) | | 0.13 (0.0098, 1.8) | 10 (0.11, 910) |
| Age | | 1.1 (0.95, 1.2) | 1.1 (0.96, 1.3) |
| Sex | | 8.5 (1.9, 38) | 4.7 (0.49, 45) |
| Group A x Study Day | | 10 (0.51, 210) | 0.019 (0.00011, 3.4) |
| Group B x Study Day | | 1.5 (0.096, 22) | 0.012 (0.00012, 1.2) |
| Group D x Study Day | | 4.7 (0.0035, 6300) | 0.027 (3.7e-07, 1900) |

†. Group A: Nasal Inoculation with influenza A/Wisconsin/67/2005; Group B: Symptomatic with T>37.8˚C or positive antigen test; Group C: Surveillance cohort with any symptoms and positive PCR; Group D: Symptomatic, seeking medical care.

‡. We used linear mixed-effects models for censored responses (R Project package “lmec”) to model the effect of groups on viral shedding over time. These models accounted for the censored outcome variable as well as nested random effects of individuals and samples within the same individuals. The best models were selected based on Akaike Information Criterion (AIC). None of the interaction terms demonstrated a significant effect.

§. Study Day was defined as day post symptom onset for Group B, C, and D, and day post inoculation minus two days for Group A. We included only those samples whose study day was not missing (three C and one D cases were excluded due to not having symptom onset date on file or having no symptoms over the course of the follow-up period).

## Supplementary Figure

**
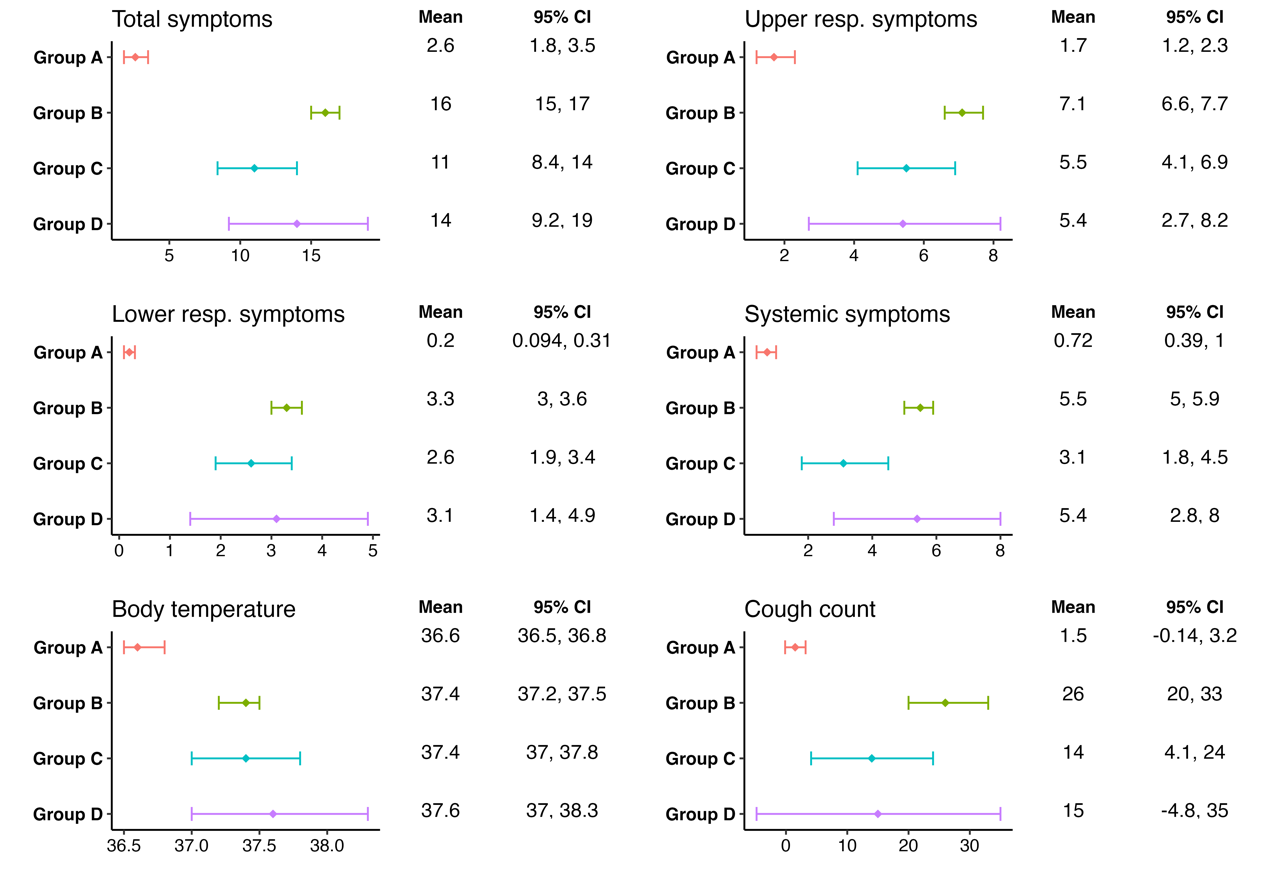
**

### **Supplementary Figure 1. Mean symptom scores, body temperatures, and cough counts across groups.**

Group A: Nasal Inoculation with influenza A/Wisconsin/67/2005; Group B: Symptomatic with T>37.8˚C or positive antigen test; Group C: Surveillance cohort with any symptoms and positive PCR; Group D: Symptomatic, seeking medical care.
